# HPB Surgery: An Independent Speciality or a Branch of Digestive Surgery?*

**DOI:** 10.1155/1994/18508

**Published:** 1994

**Authors:** Giuseppe Gozzetti, Alighieri Mazziotti, Gian Luca Grazi

**Affiliations:** 2nd Department of Surgery University of Bologna Sant'Orsola Hospital Via Massarenti, 9 Bologna 40138 Italy


*HPB Surgery*, 1994, Vol. 8, pp. 111–113
Reprints available directly from the publisher
Photocopying permitted by license only

© 1994 Harwood Academic Publishers GmbH
Printed in Malaysia

### HPB Surgery: An Independent Speciality or a Branch of Digestive Surgery?*

GIUSEPPE GOZZETTI, ALIGHIERI MAZZIOTTI and GIAN LUCA GRAZI

2nd Department of Surgery, University of Bologna, Sant'Orsola Hospital, Via Massarenti, 9, 40138 Bologna, Italy

List of the surgeons who responded to the questionnaire and conveyed their personal comments or suggestions:
Ballesta Lopez, C. (Spain), Belghiti, J. (France), Belli, L. (Italy), Berard, P. H. (France), Bismuth, H. (France), Boillot, O. (France), Boissel, P. (France), Bresadola, F. (Italy), Broelsch, C. E. (Germany), Calleja Kempin, I. J. (Spain), Calne, R. (United Kingdom), Castagneto, M. (Italy), Castro Sousa, F. (Portugal), Chapuis, Y. (France), Chipponi, J. (France), Cortesini, R. (Italy), Cuilleret, J. (France), D'Amico, D. (Italy), de Hemptinne, B. (Belgium), Decurtins, M. (Switzerland), Descottes, B. (France), Di Carlo, V. (Italy), Eigler, F. W. (Germany), Ericzon, B. G. (Sweden), Fagniez, P. L. (France), Fékété, F. (France), Foster, J. (USA), Fourtanier, L. (France), Franco, D. (France), Funovics, J. M. (Austria), Galmarini, D. (Italy), Gigot, G. F. (Belgium), Gennari, L. (Italy), Habib, N. A. (United Kingdom), Hayter, B. (United Kingdom), Houssin, D. (France), Jeppsson, B. (Sweden), Kalicinski, P. (Poland), Karlberg, J. (Sweden), Kriste, G. (Germany), La Calle, P. (Spain), Lambiliotte, J. P. (Belgium), Launois, B. (France), Li, A. (Hong Kong), Lygidakis, N. (Greek), Maffei Faccioli, A. (Italy), Makuuchi, M. (Japan), Margreiter, P. (Austria), Mathisen, O. (Norway), McMaster, P. (United Kingdom), Meurisse, M. (Belgium), Moreno Gonzalez, E. (Spain), Mouiel, J. (France), Nagasue, N. (Japan), Nagorney, D. M. (USA), Neuhaus, N. (Germany), Nuzzo, G. (Italy), Obertop, H. (Holland), Otte, J. B. (Belgium), Paquet, K. J. (Germany), Penninckx, F. (Belgium), Pichlmayr, R. (Germany), Pisa, F. (Austria), Puntis, M. C. A. (United Kingdom), Rohner, A. (Switzerland), Rossi, R. (USA), Rovati, V. (Italy), Scheele, J. (Germany), Segol, P. (France), Slooff, M. (Holland), Starzl, T. E. (USA), Tiberio, G. (Italy), Tobe, T. (Japan), Williamson, R.C.N. (United Kingdom).

Surgery began to separate into individual specialities more than 30 years ago when Urology, Vascular and Plastic Surgery became independent entities in Europe. Today, no one doubts the value of these divisions in view of the improved results obtained with patient management by specialist teams. The advantages are also evident in the research field, due to the numerically larger and more homogeneous series available with more rigorous follow-up protocols.

Currently, General Surgery is synonimous with Digestive Surgery. However, the techniques acquired during basic "general" surgical training (including Thoracic, Vascular and Urological Surgery) are fundamental to the success of emergency or complex major procedures, and also for surgical practice in peripheral hospitals where the specialities are not available.

Over the past 10 years, Hepato–Pancreatic–Biliary (HPB) Surgery has rapidly developed. Advances in diagnostic procedures were the first factors to contribute to this development. It is now possible to diagnose tumors at an earlier stage allowing accurate planning of operative strategy and thus improving the chances of complete removal. At the same time an actual increase in the frequency of HPB tumors has been reported in the literature, especially hepatic tumors. Another factor in the development of HPB surgery is collaboration with non-surgical specialists: Gastro- enterologists, Radiologists, and Endoscopists increas- ing the therapeutic options for otherwise inoperable patients. The final important factor in the expansion of HPB Surgery is the clinical impact of liver and pancreas transplantation.

* Lecture given at the 1st European Congress of HPB Surgery, Paris, June 8–11, 1993

The emergence of the scientific and research aspects of HPB Surgery has seen the foundation of societies, such as International Hepato–Biliary–Pancreatic Association and the World Association of Hepato–Pancreato–Biliary Surgery founded in Lund in 1986. With these societies have come more frequent meetings in this subject area. Scientific publications relating to HPB Surgery represent a large proportion of the articles accepted by General Surgery journals. During 1992, 378 articles on HPB Surgery, were among the total number of 1755 published in 8 different General Surgery journals*.

This activity over recent years has been associated with the creation of specialized HPB Units throughout Europe. The first examples of this move were by Stig Bengmark in Lund and Henry Bismuth in Paris. Their work provided the impetus for the creation of a HPB Surgery specialization. We have a clear demonstration of how the creation of a HPB unit can be of advantage when we consider their results in terms of clinical outcome, the scientific value of their numerically substantial studies, the accurate follow-up they have achieved, and lastly, the experimental studies that have been carried out. Their example has been followed by other European Centers, especially following the success of liver transplants.

Nevertheless, we are still far from accepting the separation of HPB Surgery from Digestive Surgery. Fragmentation in surgery, the possible limited knowledge of young surgeons, increasing institutional costs for more isolated and "specialist units", and lastly, a small number of patients, especially in certain geographical areas, are all factors against the separation of HPB Surgery.

Today the necessity for a statement about where HPB Surgery is going is needed and we have tried to determine whether or not the time has come for HPB Surgery to become an independent speciality from Digestive Surgery.

We have not yet found a conclusive answer to this question and we do not want to take a stand for or against the separation of HPB Surgery from Digestive Surgery. Our aim in this paper will be to present a picture of the current European situation and report the opinions from surgeons in several countries. These opinions have come from a questionnaire sent to 136 Centers in 17 European countries and also for comparison, to 3 extra-European Centers. For the most part these were recognized Digestive Surgery Centers where HPB Surgery is carried out. The others were specialized Centers for HPB Surgery. We enquired about the organization of wards, frequency of HPB Surgery, educational and research activities, and lastly, personal opinions on the separation of HPB Surgery into a specialism.

We received 70 completed questionnaires and 4 surgeons only sent in comments and suggestions, all were considered in the final analysis. Sixty four (91.4%) of the answers came from University Centers. Thirteen (18.6%) of the Centers which responded were HPB units, while the remaining 57 (81.4%) were Digestive Surgery Departments.

The first group of questions concerned the impact of HPB surgery: the number of cases seen per year, the specific type of surgical procedures performed, and the organization of the ward. An average of 20.2 (range 8–60) beds were reserved for HPB diseases either in HPB Units or in Departments of Digestive Surgery.

The total number of HPB procedures performed during 1992 did not differ between HPB Units and Digestive Surgery Departments. In the latter, HPB procedures accounted for 23.9% of the total number of operations performed. The number of transplants carried out in the two different settings were also similar (24.9 in 1992).

These data indicate that the demand of HPB Surgery is significant throughout European Countries. Quantitatively the amount of work is remarkable both in specialist units and in Digestive Surgery Centers. Also qualitatively, activity in this type of surgery is considerable, especially with an increasing number of liver transplants being carried out. A higher number of transplants were carried out in specialist HPB Units, 34.7 versus 22.7 in Digestive Surgery Departments. Besides this, there were no other differences as to the type of surgical procedures carried out in the specialist HPB Units and Digestive Surgery Departments. The number of laparoscopic cholecystectomies was slightly greater in the Digestive Surgery Departments.

A peculiar aspect of organization that came out of our study is that in 56.1% of Digestive Surgery Centers also performing HPB surgery, 7.8 surgeons were appointed to manage only HPB Surgery patients. This trend toward the creation of a specialized team of surgeons was seen even more clearly when it came to transplant activity: in the majority of cases (66.7%), this activity was carried out by specialized teams whereas in only 20.7% of cases, the activity was shared by all the surgeons in the Center. According to the responses that we received, there is a tendency to

* American Journal of Surgery; Annals of Surgery; Archives of Surgery; British Journal of Surgery; Journal de Chirurgie, Surgery; Surgery, Gynecology & Obstetrics, World Journal of Surgery.

consider HPB Surgery as a very specialized field that requires well-trained surgeons with specific technical and medical competencies. This trend is even more evident in the area of liver transplantation.

Forty four percent of the Centers had specific outpatient clinics for HPB diseases. The HPB units had a larger percentage of specific outpatient clinics with 76.9% versus 36.4% in the Digestive Surgery Department. In consequence a larger number of patients could be seen and followed-up in HPB units. There was no difference when looking at related surgical activities amongst Digestive Surgery Departments and HPB units: Interventional Radiology, Medical Oncology, and Operative Endoscopy were routinely performed by Digestive and HPB surgeons.

The second group of questions concerned the training, residency, and experimentation programs. 54.5% of colleagues who responded to the questionnaire did not favor the creation of specific residency programs in HPB Surgery. Instead, 73.8% were in favor of the institution of post-residency courses or fellowship programs in HPB Surgery.

HPB experimental activity was practiced on a regular basis in 59.7% of the responding Centers. As expected, the activity was more widely seen in HPB units (76.9%) than in Digestive Surgery Departments (55.6%). This shows how the institution of such units could provide training for young surgeons in terms of experimental and clinical research, and specialist management of patients.

The last question in the questionnaire asked specifically whether or not HPB Surgery should be considered a surgical speciality. Only 19 (27.5%) were in favor of the institution of a new HPB speciality, and our personal feeling is decisively amongst them.

An analysis of the answers to this final question seen in the light of the preceeding responses showed some intriguing aspects.

As expected, 61.5% of the Centers where an HPB unit was already established, answered in favor of the creation of an independent speciality. On the contrary, the large majority (80.4%) of surgeons practicing within a Digestive Surgery Department did not think that HPB Surgery should be considered a separate new speciality.

The answers were equally negative in most of the Centers whether or not they performed liver transplants. Furthermore, 77.8% of the Centers where more than 60 liver transplants were performed during 1992, and where HPB activity was carried out with a greater frequency, expressed a negative opinion about the creation of a separate HPB speciality. Surgeons are concerned to keep HPB Surgery within the sphere of Digestive Surgery which is its natural location both culturally and practically.

On the other hand, 56% of those who responded negatively to the constitution of a HPB speciality had already established, within their own Digestive Surgery Departments, specialized teams for HPB surgery and/or liver transplants. To us this proved that the need for a separation of HPB Surgery, which could improve clinical results, is actually already present. In our opinion, liver transplant activity is the major impulse toward the autonomy of HPB Surgery.

In conclusion, it can be said that in the '90s a full maturation of HPB Surgery is taking place as a result of the frequency of common hepato-biliary diseases and the complexity of either surgical therapy or the complementary or alternative treatment to surgery. The number of scientific publications and meetings that every year address the subject of HPB Surgery, proves the extent of involvement in this field and is similar if not greater than the number present in other surgical specialities that have had autonomy of years.

From the analysis of the questionnaire, the recognition of cultural and scientific maturity is evident. Likewise, the questionnaire supports the trend present throughout Europe, to either create a HPB Surgery unit or form a specialized HPB team which operates within a Digestive Surgery Department. In spite of all the reasons which support the autonomy of HPB Surgery, the predominant opinion amongst European surgeons is against its separation from General Surgery out of which HPB Surgery has grown. Obviously, it is inconceivable to think of a Hespato–Biliary surgeon who is not familiar with the fundamentals of Digestive Surgery (i.e. enteric anastomoses, digestive physiopathology, bilio and pancreatic-enteric anastomoses, etc.). Likewise, in order to practice HPB Surgery, the fundamentals of Vascular and Thoracic Surgery are considered indispensable. Probably the complexity of HPB Surgery and other connected activities such as Diagnostic and Operative Radiology, Endoscopy, and Oncology, entail a new and broader concept of a speciality which could be thought of as a development of Digestive Surgery, which remains the foundation of Hespato–Biliary Surgery training.

We personally believe that rather than a division, the future will see the progression of Digestive Surgery to include advanced Hespato–Biliary Surgery with the institution of multidiciplinary units which bring together Endoscopists, Radiologists, and Gastroenterologists thus giving the surgeon a preeminent coordinating role in achieving a more rational approach to the treatment of patients.

